# Methodological Quality of Pulmonary Arterial Hypertension Treatment Evidence-Based Guidelines: A Systematic Review Using the AGREE II and AGREE REX Tools

**DOI:** 10.1007/s10557-024-07605-w

**Published:** 2024-07-09

**Authors:** Ana Paula Oliveira Vilela, Flávia Deffert, Rosa Camila Lucchetta, Yara Maria da Silva Pires, Felipe Fernando Mainka, Fernanda S. Tonin, Roberto Pontarolo

**Affiliations:** 1 Pharmaceutical Assistance Postgraduate Program, Federal University of Paraná, Curitiba, Brazil; 2 Pharmaceutical Sciences Postgraduate Program, Universidade Federal Do Paraná, Curitiba, Brazil; 3 Health Technology Assessment Unit, Oswaldo Cruz German Hospital, São Paulo, Brazil; 4 Pharmaceutical Sciences Postgraduate Program, Federal University of Paraná, Curitiba, Brazil; 5 H&TRC-Health & Technology Research Center, ESTeSL-Escola Superior de Tecnologia da Saúde, Instituto Politécnico de Lisboa, Av. Dom João II Lote 4.69 01, 1990-096 Lisbon, Portugal; 6 Department of Pharmacy, Federal University of Paraná, Curitiba, Brazil

**Keywords:** Practice guideline, Quality assessment, Pulmonary arterial hypertension, AGREE II, AGREE REX

## Abstract

**Purpose:**

Pulmonary arterial hypertension (PAH) is a progressive disease with a poor prognosis, and its management should be grounded in well-developed clinical practice guidelines (CPG). Thus, we critically assess the methodological quality of the available CPG for pharmacological treatments for PAH.

**Methods:**

A systematic review (CRD42023387168) was performed in PubMed, Cochrane, Embase, and Tripdatabase (Jan-2023). Eligible records were appraised by four reviewers using the Appraisal of Guidelines, Research, and Evaluation Collaboration tool (AGREE II) and the complementary tool for assessing recommendations’ quality and certainty, AGREE REX. Descriptive statistics were used to summarize the data.

**Results:**

Overall, 31 guidelines, mainly authored by professional societies (90%), targeting only physicians as primary users (84%), were identified. Guidelines presented a moderate overall quality (scores of 63% and 51% in AGREE II and AGREE REX, respectively), with a few domains showing slight improvements over the years. AGREE II “Scope and Purpose” (94%) and “Presentation Clarity” (99%) domains obtained the highest scores. The items related to “Stakeholder involvement,” “Editorial independence,” and “Clinical applicability” (AGREE REX) were fairly reported. Conversely, CPG lacks rigor in development (32% score, AGREE II), scarcely discusses the role of stakeholders, and provides deficient data on the implementation of recommendations (scores of 35% and 46% in AGREE II and AGREE REX, respectively). No differences in the quality of guidelines published by different developers or countries were observed (*p* > 0.05).

**Conclusion:**

Methodological weaknesses are common among guidelines addressing PAH treatment, especially regarding scientific rigor, stakeholders’ values and preferences, and facilitators and barriers to implementability. Particular attention should be given to developing future guidelines.

**Supplementary Information:**

The online version contains supplementary material available at 10.1007/s10557-024-07605-w.

## Introduction

Pulmonary arterial hypertension (PAH), a subtype of pulmonary hypertension (i.e., group 1), is hemodynamically defined as a mean pulmonary arterial pressure > 20 mmHg, a pulmonary vascular resistance > 2 Wood units, and a pulmonary artery wedge pressure ≤ 15 mmHg at right heart catheterization [1]. It is a rare, highly complex, and progressive disorder that can lead to right ventricular failure and ultimately premature death [2–4]. The prevalence of PAH is around 15 to 50 cases per million within the USA and Europe, with an incidence of approximately five cases per population of 1 million per year, mainly affecting individuals between 30 and 60 years old [5, 6]. However, the triggering etiology of PAH pathogenesis is likely multifactorial (i.e., including inappropriate angiogenesis, metabolic derangements, DNA damage, and impaired vasoreactivity), idiopathic, heritable, and anorexigenic-induced PAH, which make up around 53% of the diagnosis [7–9].

Despite the remaining challenge to diagnose and treat, findings from recent clinical studies have shifted how PAH is managed—by adding further approved target drugs (yet at a remarkably high cost) to the symptomatic treatment to reduce right ventricle afterload. These practices are now reflected in recently revised clinical practice guidelines (CPG) jointly developed by international societies (e.g., the European Society of Cardiology (ESC)/European Respiratory Society (ERS)) [3, 8, 10]. CPGs are systematically developed statements built upon evidence-based principles and intend to provide trustworthy information with reduced risk of bias to aid practitioners and decision-makers about appropriate care and health policies within a specific clinical scenario [11]. Nevertheless, as their conduct and report may vary widely, appraisal tools have been developed to ensure that a minimum set of methodological quality features, including guidelines’ development, context, content, and application, are followed [12–14].

The refined and currently used version of the Appraisal of Guidelines, Research, and Evaluation Collaboration tool (AGREE II), published in 2010, includes 23 quality items within six domains (scope and purpose, stakeholder involvement, rigor of development, clarity of presentation, applicability, and editorial independence) [15–19], while the complementary tool for assessing recommendations’ quality and certainty (AGREE REX, Recommendations Excellence, published in 2020) comprises nine items within three domains (clinical applicability, values and preferences, local application and adoption—implementability) [17, 20]. Although these validated approaches are widely used [18], the available literature on systematic evaluations of CPGs using both tools (AGREE II and AGREE REX) is still limited to some clinical conditions [20–24].

Considering the increased number and recently updated guidelines addressing PAH treatment, a more comprehensive evaluation of their quality and generalizability is needed to identify potential weaknesses and strengths that may guide the development and improvement of future documents and practical frameworks for managing this rare disease—including new treatments’ prioritization, access, and use in practice. Thus, our goal was to critically appraise the methodological quality of CPG on pharmacological treatments for PAH using a broad systematic review and AGREE II and AGREE REX assessments.

## Methods

A systematic review was conducted following the recommendations of Joanna Briggs and the Cochrane Collaboration [25, 26] and reported following the updated PRISMA (Preferred Reporting Items for Systematic Reviews and Meta-Analyses) [27]. The protocol is available at PROSPERO (CRD42023387168) and OSF platform (10.17605/OSF.IO/E9DQ3).

### Search Strategy and Selection of Guidelines

A systematic search was performed in PubMed, Cochrane, Embase, and Tripdatabase electronic databases (updated 01/2023) using terms related to “practice guideline” and “pulmonary arterial hypertension” combined with Boolean operators AND and OR. Searches were limited (i.e., by filters) to registers published after 2012 (given the last update of the PAH treatment algorithm, including all available therapeutic approaches) (see complete search strategy in Supplementary Information). A manual search was also performed in the reference lists of the included studies and websites of governmental and non-governmental organizations (Australian Clinical Practice Guidelines, Brazilian Ministry of Health, Canadian Agency for Drugs and Technologies in Health, Chilean Ministry of Health, Colombian Ministry of Health and Social Protection, Guidelines International Network (GIN), Institute for Clinical Systems Improvement, Portal Guía Salud, Scottish Intercollegiate Guidelines Network, and the National Institute for Health and Care Excellence, WHO, and other governmental or non-governmental websites). All other documents (e.g., appendix, development guidance, declaration of conflict of interests) related to the eligible guidelines were manually obtained (when available) from the sources described in the main report.

Studies were included if they met the following eligibility criteria: designed as a CPG including explicit recommendations (i.e., statements from an expert panel based on the best evidence) on the pharmacological treatment (i.e., drugs at any dose, regimen, or combination) of patients diagnosed with PAH. Only documents available online and authored by professional societies or governmental organizations were considered for eligibility. During the screening (title and abstract reading) and full-text eligibility phases, articles were excluded if they were considered irrelevant to the study goals. Exclusion criteria are as follows: records written in non-Roman characters.

### Data Extraction and Guideline Assessment

Retrieved records were imported into Rayyan [28] for duplicate removal, screening, and full-text appraisal. Two authors did all study selection and data extraction steps independently, with a third author to resolve discrepancies. Excel spreadsheets were used to extract guidelines’ data on overall information (i.e., organization or governmental parties involved, publication year, geographic location, and evidence grading tools).

The methodological appraisal of the included guidelines (methods, evidence synthesis, and recommendations) was performed independently by four reviewers (APV, FD, FM, YMP), using both AGREE II, 23 quality items, and AGREE REX, a nine-item instrument. Disagreements were resolved by discussion among referees. Both tools were evaluated using a 7-point Likert scale: 1 = strongly disagree (poor or no fulfillment of the criteria) and 7 = strongly agree (all criteria were disclosed entirely). The domains’ grades were calculated as described in the users’ manual ((obtained score − minimum score possible)/(maximum score − minimum score)), and the overall assessment was calculated through the mean of the respective instrument domains. The final rate was achieved through a consensus of instruments’ domain grades between the evaluators. Results were expressed in percentages (standardized scores for each domain range from 0 to 100%).

### Data Analysis and Synthesis

Descriptive statistics were used to summarize the data. The Kolmogorov–Smirnov test assessed data normality with additional visual inspection of the QQ plots, confirming that the data was non-normal. For dichotomous variables, absolute and relative frequencies were reported, while continuous variables were expressed in the median, interquartile range (IQR), and minimum–maximum values.

Subgroup differences were explored using the Whitney test for overall and domain scores regarding the type of CPG developer (i.e., professional or governmental institutions) and publication date (categorized as before or after the median of the publication date). The Kruskal–Wallis test was used to compare general scores and domains between continents, following the geographical origin of publications. The differences’ sizes were evaluated using the Monte Carlo method with 95% confidence intervals (95% CI) as effect size measures. Spearman’s correlation was used to assess the relationship between the distinct domains from the AGREE II and AGREE REX tools. The intraclass correlation coefficient (ICC) with a two-way mixed effects model was used to verify interrater agreement based on the 95% CI of the ICC estimate (values less than 0.5, between 0.5 and 0.75, between 0.75 and 0.9, and greater than 0.90 are indicative of poor, moderate, good, and excellent reliability, respectively) [29]. Analyses were performed in IBM SPSS Statistics v.28, with a significance level set at 0.05.

In addition to the statistical analysis, an informal analysis was carried out by visual inspection of the graphs, considering recommendations from the PAH guidelines. In this analysis, only recommendations focused on target therapy for PAH were considered, according to the risk classification (low, intermediate, or high risk).

## Results

### Overall Characteristics of the Guidelines

**Fig. 1:**
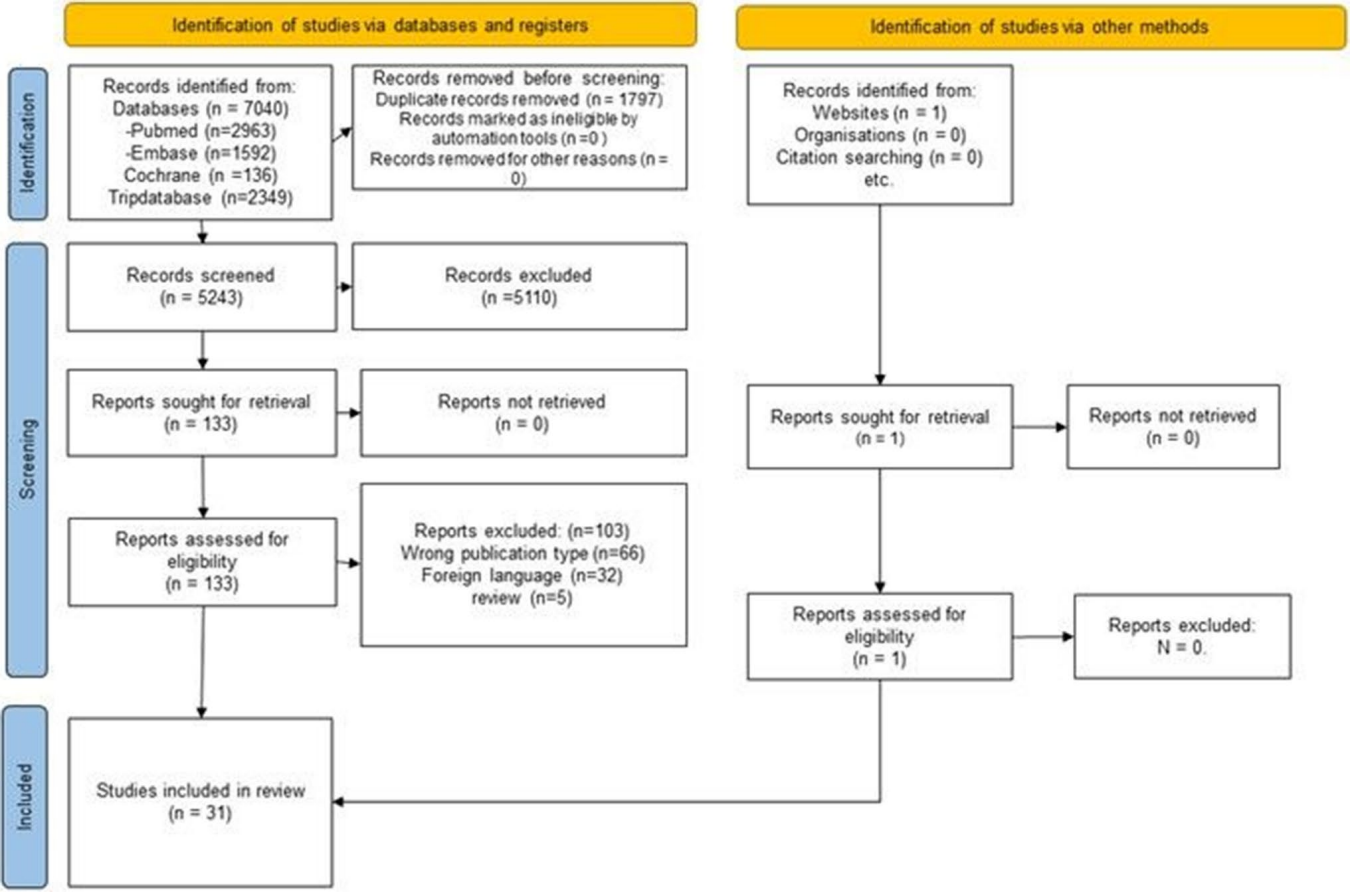
Systematic review process

A total of 5243 registries were obtained after duplicate removal through database searches. During the screening process (title/abstract reading), 5163 records were considered irrelevant to the study. The remaining 80 had their full-text appraised, of which 50 were excluded as they did not meet the eligibility criteria (full exclusion details are shown in Supplementary Information). One document was found by manual search (website), totaling 31 guidelines for synthesis (see Fig. 1).

These 31 guidelines were published between 2012 and 2022, with 2018 being the median year of publication. Most documents are from North America (*n* = 14; 45.2%), followed by Asia (*n* = 7; 22.6%) and Europe (*n* = 6; 19.4%). South America and Africa published three (9.7%) and one (3.2%) guideline. Most guidelines (*n* = 28; 90.3%) were published by professional societies; only three (9.7%) PAH-MS 2014, CHINA 2020, PROSTACYCLIN were developed by governmental institutions (references to the guidelines can be found in Supplementary Information). Of the 31 guidelines, only five—ATS 2014, CHEST 2014, AHA-ATS 2015, CHEST 2019, ESC ERS 2022—(16%) followed the recommendations of GRADE—Grading of Recommendations Assessment, Development and Evaluation—to evaluate the evidence and strength of recommendation, and three guidelines—CCS-CTS 2020, KSC-KATRD 2020, HAT 2021—(10%) followed AGREE for more careful elaboration of the guidelines. Regarding the development of recommendations, ten guidelines (32%) used expert consensus, another ten (32%) carried out systematic reviews, and eleven (36%) were based on other previously published documents. The disease is associated with high treatment costs, and using resources for the healthcare system is a significant challenge; 29 guidelines (94%) did not report on this issue. The main characteristics of the included CPG are available in Supplementary Information. ICC among the four reviewers for the assessment of AGREE II and AGREE REX was 0.769 (95% CI, 0.722–0.811) and 0.578 (95% CI, 0.480–0.672), showing good and moderate overall interrater agreements, respectively.

### AGREE II

The median overall AGREE II score for the 31 CPG was 63 (IQR 58–76), with domains 1, 2, 4, and 6 presenting higher scores. Most guidelines (*n* = 20; 64.5%) obtained a moderate-to-high quality overall score (50–75%), while 3 (9.7%) were judged to have low-moderate quality (25–50%) and 8 (25.8%) high quality (> 75%). No guidelines obtained an overall low-quality score (< 25%) (see Table 1).

**Table 1:**
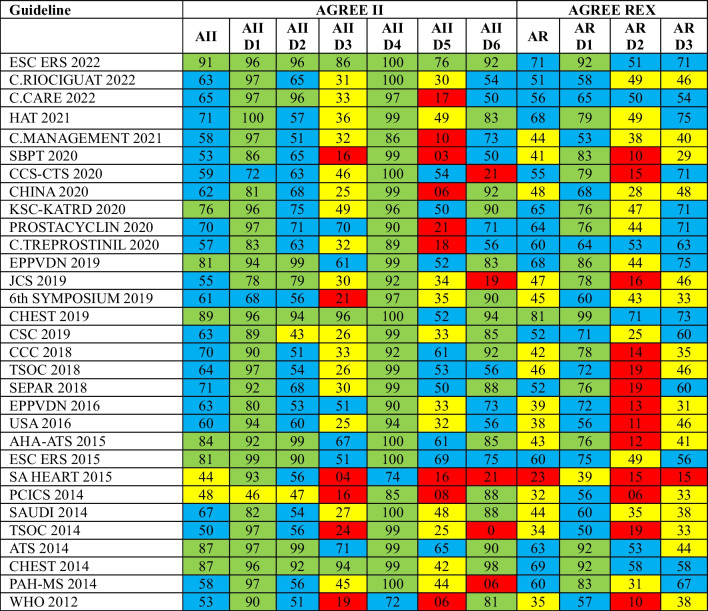
AGREE II and AGREE REX scores of the 31 PAH’ treatment guidelines

AGREE II domains: (AII) global assessment; (AII D1) domain 1—scope and purpose; (AII D2) domain 2—stakeholder Involvement; (AII D3) domain 3—rigor of development; (AII D4) domain 4—clarity of presentation; (AII D5) domain 5—applicability; (AII D6) domain 6—editorial independence

AGREE REX domains: (AR) global assessment; (AR D1) domain 1—clinical applicability; (AR D2) domain 2—values and preferences; (AR D3) domain 3—implementability

Note: Green boxes refer to high-quality scores (above 75%), blue boxes for moderate-high quality scores (between 50 and 75%), yellow boxes at low-moderate quality indices (between 25 and 50%), and red boxes for low-quality scores (below 25%)

Abbreviations: *AHA-ATS* American Heart Association and American Thoracic Society, *CCC* Cologne Consensus Conference, *EPPVDN* European Pediatric Pulmonary Vascular Disease Network, *ESC\ERS* European Society of Cardiology and European Respiratory Society, *JCS* Japanese Circulation Society, *PCICS* Pediatric Cardiac Intensive Care Society, *SAUDI* Saudi Arabia, *TSOC* Taiwan Society of Cardiology, *6th Symposium* 6th World Symposium on Pulmonary Hypertension, *ATS* American Thoracic Society, *SBPT* Brazilian Society of Pulmonology and Phthisiology, *CCS-CTS* Canadian Cardiovascular Society-Canadian Thoracic Society, *CHEST* Chest Guideline and Expert Panel Report, *CHINA* Chinese expert-based consensus, *CSC* Sociedad Colombiana de Cardiología, *USA* United States of America, *PAH-MS* Pulmonary Arterial Hypertension–Ministry of Health of Brazil, *HAT* Heart Association of Thailand, *KSC-KATRD* Korean Society of Cardiology and the Korean Academy of Tuberculosis and Respiratory Diseases, *SA HEART* South African Heart Association, *SEPAR* Spanish Society of Pulmonology and Thoracic Surgery, *C.Treprostinil* Consensus Treprostinil, *C. Riociguat* Consensus Riociguat, *C. Management* Consensus Management, *C.Care* Consensus Care, *WHO* World Health Organization

In domain 1 (scope and purpose), the median score was 94 (IQR 83–97), with all guidelines scoring above 68%, except PCICS 2014 (a North American pediatric guideline developed by a professional society) with 46%. The Thai Guideline (HAT) 2021 received the highest score in this domain by meeting 100% of the required criteria. Similarly, high overall scores were obtained in domain 4 (clarity of presentation) (median 99 (IQR 92–100) with almost all documents scoring above 85%) and in domain 2 (stakeholder involvement; median score 63 (IQR 54–90)). Although most guidelines were judged as having moderate-to-high quality in domain 6 (editorial independence) (overall 81 (IQR 54–90)), a critical dispersion exists—with scores ranging from 0% (six guidelines scoring < 25%) to 98%.

Although three guidelines (Chest 2014 and Chest 2019 from North America and the European ESC ERS 2022) were judged as having high quality in domain 3—rigor of development (> 85% scores), around one-third of CPG presented low quality—an overall median of only 32 (IQR 25–51), the SA HEART 2015 guideline from Africa fulfilled only 4% of the criteria of this domain. Equally poor results were found for domain 5 (applicability) with a median score of 35 (IQR 18–52); nine guidelines (29.0%) presented low quality. The European ESC ERS 2022 was the only guideline to obtain scores > 75% in this domain.

**Fig. 2:**
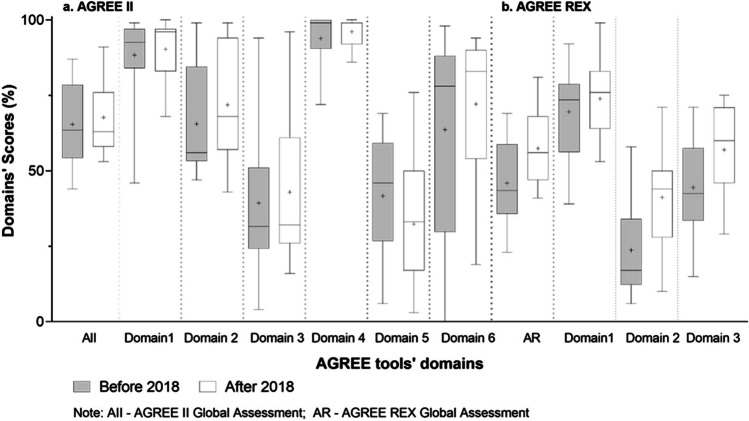
AGREE II and AGREE REX results according to guidelines’ date of publication. AGREE II domains: domain 1—scope and purpose; domain 2—stakeholder involvement; domain 3—rigor of development; domain 4—clarity of presentation; domain 5—applicability; domain 6—editorial independence; AGREE REX domains: domain 1—clinical applicability; domain 2—values and preferences; domain 3—implementability

**Fig. 3:**
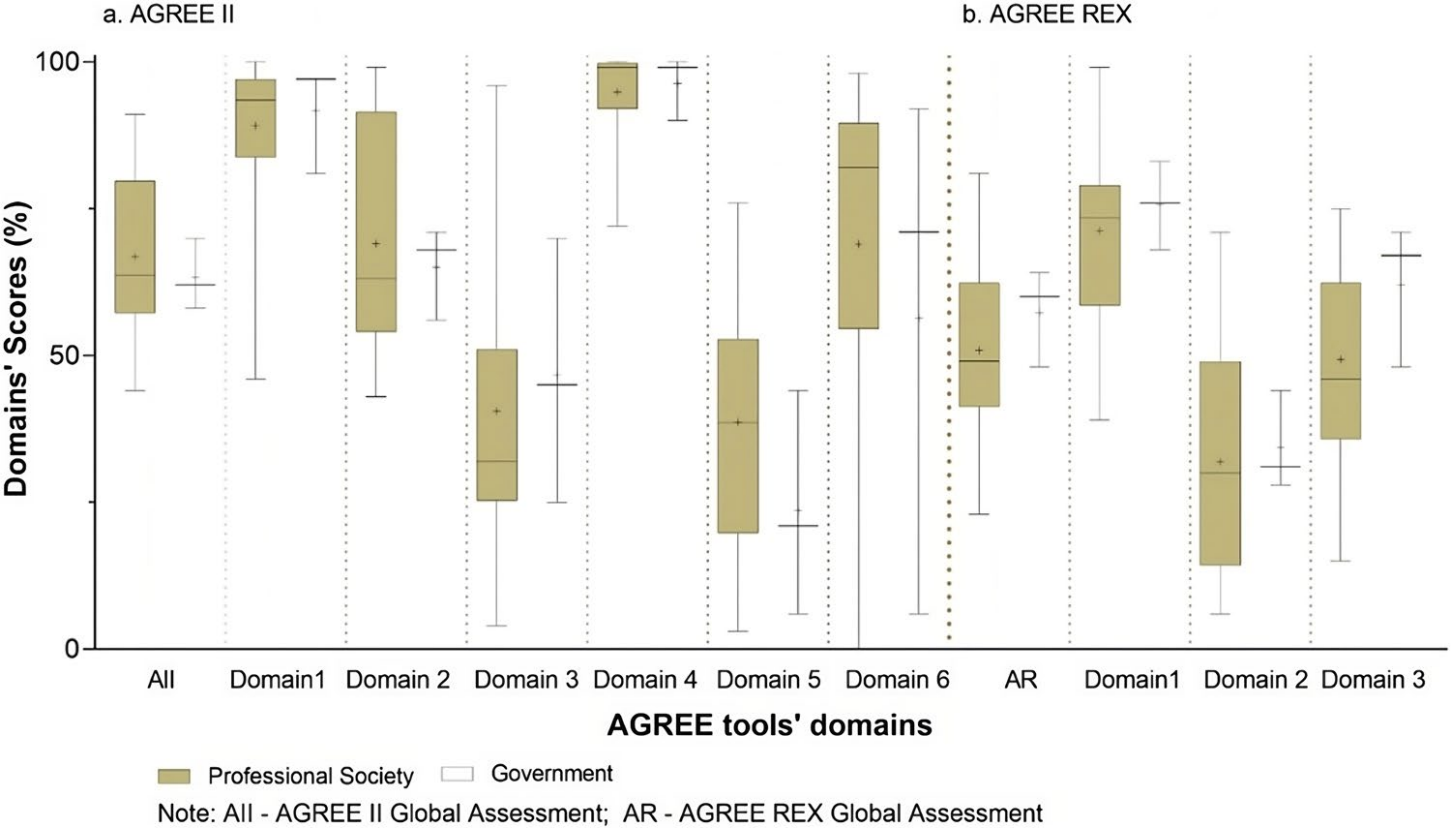
AGREE II and AGREE REX results according to guidelines’ developers. AGREE II domains: domain 1—scope and purpose; domain 2—stakeholder involvement; domain 3—rigor of development; domain 4—clarity of presentation; domain 5—applicability; domain 6—editorial independence; AGREE REX domains: domain 1—clinical applicability; domain 2—values and preferences; domain 3—implementability

Despite these slight differences, no significant changes in CPG scores were observed over the years (Fig. 2). Similar scores were also obtained for guidelines published either by governmental bodies or professional societies (Fig. 3) and by documents from different countries (geographical regions) (see Supplementary Information for additional figures and complete analyses).

### AGREE REX

The median overall AGREE REX score for all included CPG was 51 (IQR 42–63), with most guidelines (*n* = 15; 48.4%) presenting moderate-to-high quality, followed by those with low-moderate quality (*n* = 14; 42.2%). Low-quality and high-quality scores were found for one guideline each. Nonetheless, guidelines scored better only in domain 1 (clinical applicability) with a median result of 75.0 (IQR 60–79) and no CPG with low quality in this domain. Median scores in domains 2 (values and preferences) and 3 were 31.0 (IQR 15–49) and 46.0 (IQR 38–67), respectively, with 6 and 14 guidelines (mainly from North America and Europe) achieving scores between 50 and 75% in these domains, respectively (see Table 1).

Yet, significant improvements over time on the overall AGREE REX results were observed in the association analyses (see Fig. 2): publications before 2018 scoring 43.5 (IQR 35.8–58.8) and those after 2018 scoring 56.0 (IQR 47.0–68.0) (Mann–Whitney *U* = 59; *p* = 0.016). Similar trends were obtained for both individual domains 2 (values and preferences) and 3 (implementability) (*U* = 58; *p* = 0.014 and *U* = 65; *p* = 0.029, respectively) of this tool. No other significant differences according to guidelines’ developers (governmental or society; Fig. 3) nor geographical origin were observed (see Supplementary Information for additional figures and complete analyses).

Finally, the correlation matrix (see Supplementary Information) confirmed that similar patterns between AGREE II and AGREE REX exist, with a high correlation (Spearman rho = 0.722) between the overall scores of the two instruments. All individual domains have a moderate-to-high correlation with their respective tools.

**Fig. 4:**
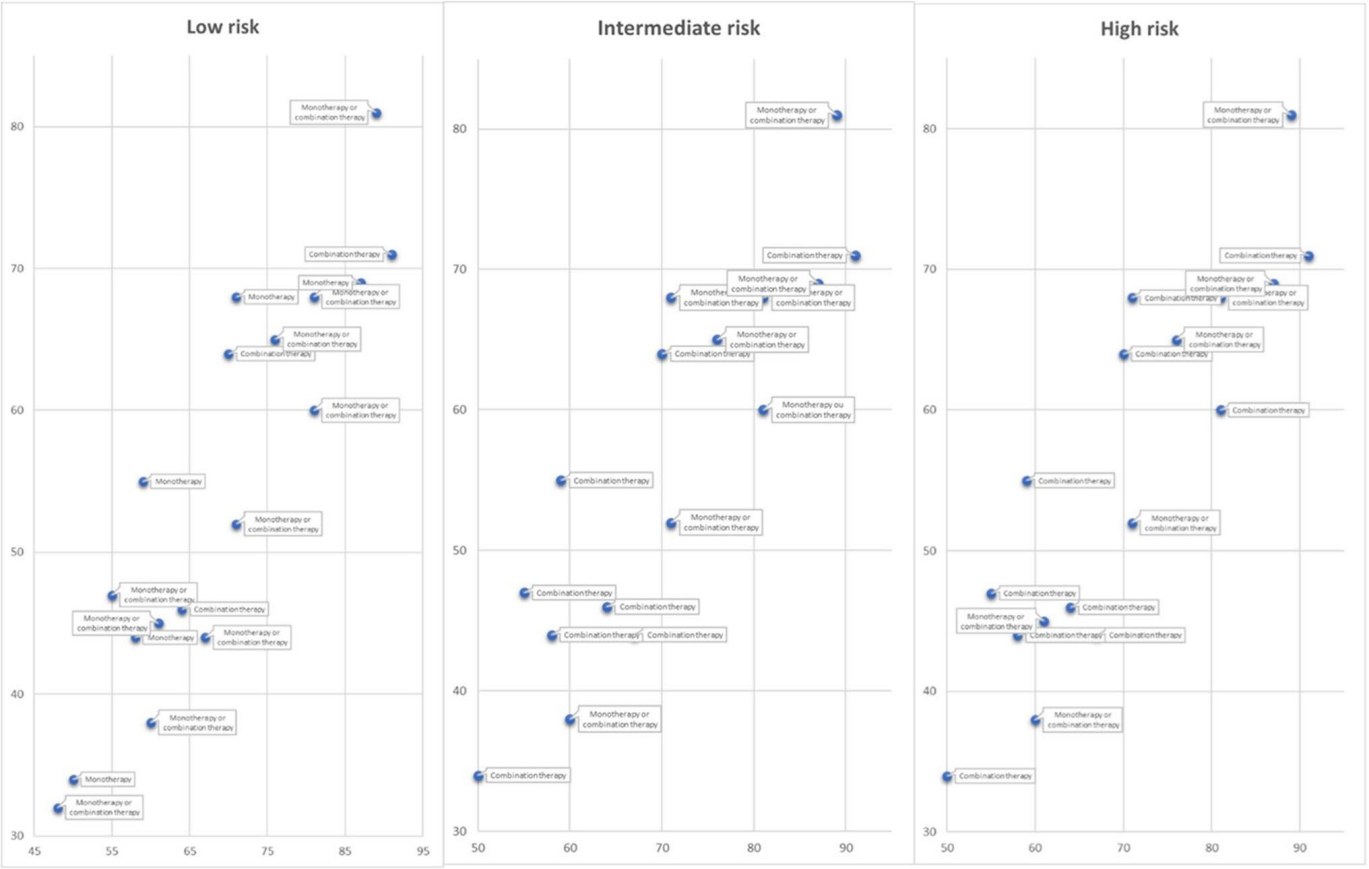
Summary of recommendations from PAH’ treatment guidelines according to AGREE II and AGREE REX scores. Only recommendations that considered the WHO functional class for risk categorization were considered. Guidelines that did not formulate recommendations according to risk categorization (not applicable) or that did not make any pharmacological recommendations explicit (not reported) were excluded from this analysis (see Supplementary Information)

No relationship between the quality of guidelines (AGREE II or AGREE REX) and the recommendations, considering the options between monotherapy and combination therapy (Fig. 4) or pharmacological classes (Supplementary Information), was found in the PAH target therapy recommendations analysis.

## Discussion

This study systematically and critically appraised the methodological quality of 31 CPG on PAH treatments published by four continents using AGREE II and AGREE REX tools. The results of these instruments correlate to the guidelines’ credibility and the validity of their recommendations. While adherence to clinical guidelines is paramount for healthcare providers and patients to improve the quality of care and achieve positive outcomes, periodic assessment of guidelines’ quality is also important, including to address situations where treatment acquisition is restricted and associated with high resource utilization and financial strain—as is often the case in PAH management (e.g., high cost of new pharmacological treatments, challenges with prior authorization, tiered use) [4, 5, 10]. Yet, as moderate-to-good agreement rates among reviewers were obtained when using the AGREE tools in our study, findings can be considered reliable and consistent in similar scenarios [17, 30, 31].

Overall, we found higher scores for AGREE II’s domains 1 (scope and purpose) and 4 (clarity of presentation) and for AGREE REX’s domain 1 (clinical applicability), which is similar to the findings reported by previous studies for other clinical conditions, including nutrition care, mental disorders, and medical oncology [21, 32–36]. These results mean a CPG document written in comprehensive language with a clear structure and presentation of goals regarding specific health issues and target populations. Guidelines written in concise, unambiguous language appear to be the most accessed by clinicians and are more likely to be implemented into clinical practice [33]. Conversely, several methodological issues were identified in domains 3 (rigor of development) and 5 (applicability) of AGREE II, and domains 2 (values and preferences) and 3 (implementability) of AGREE REX as also previously highlighted in the biomedical literature, including in areas of intensive care units, skin disorders, and lipid management for patients with coronary heart disease [21, 24, 32, 34, 35]. Insufficient scientific rigor for developing guidelines, lack of standard procedures for evidence synthesis and recommendations’ formulation, scarce information on the implementation process, and failure to consider patients’ and other target users’ opinions may negatively affect the findings of these documents. This is concerning, especially for rare diseases (such as PAH) with deficient evidence. Skewed guidelines towards researchers’ or health professionals’ values and preferences may lead to less meaningful patient guidelines and a low CPG implementation rate, contributing to knowledge waste in various clinical settings (results are inaccessible or unused) [11, 37].

Although the information on “stakeholder involvement” (domain 2 AGREE II) and “editorial independence” (domain 6 AGREE II) was fairly provided by most PAH guidelines, important pitfalls were detected on AGREE REX domains 2 and 3 (values and preferences and implementability). These findings are consistent with previous research in actinic keratosis, pressure injury, and uveal melanoma, highlighting the need to improve the proximity of clinical practice to patients’ values and characteristics, which should not be restricted to populations’ characteristics (e.g., sex, gender, race, genetic profile), and consider behavioral and social aspects and treatment costs. The involvement of other stakeholders, such as health policymakers and government representatives, is paramount for the continuous development and practical implementation of a guideline [24, 38, 39]. For this to occur, communication between CPG’s working parties, including a wide range of representatives (e.g., professional organizations, regional and local offices, and relevant national bodies), is essential. Nonetheless, there may be several barriers hampering involvement, including the identification and recruitment of appropriate stakeholders, the definition of roles and responsibilities, and disclaiming the potential conflicts of interest and resources, which should be clearly stated in the guideline (either on the main document or supplementary materials) for data transparency. Moreover, if applicability issues, including types of facilitators and barriers surrounding guideline implementation, are not adequately addressed, CPG may be ineffective in clinical practice and increase the waste of resources. O’Connor et al. [40] reported that less than 20% of the published guidelines on antenatal management of twin pregnancies were considered in implementing recommendations. The costs and budget concerns of applying the findings are equally poorly communicated by guidelines in PAH or other areas (including mental health, viral infections, and oncology), limiting the extent to which health systems can disseminate and use the evidence [41, 42].

Although there has been a significant increase in the number of CPG publications in the past years, including for PAH [43, 44], which is probably due to the approval of new treatments and the intention to improve the quality of care for patients while informing healthcare costs and reducing variability in clinical practice [43], their overall methodological quality (AGREE II scores) has not improved, reinforcing the need to strictly develop and report these documents to avoid suboptimal decision-making processes [30, 31, 44]. From a global perspective, although some countries have initiated national protocols regarding developing, appraising, and implementing high-quality CPG, standardized approaches for their production are still lacking [43]. This may partially justify the heterogeneity among PAH guidelines regarding the evidence-based methodological approaches used to build the reports. Although a panel of experts and GRADE collaboration tools are widely recommended strategies, some guidelines use alternative approaches, which may be the cause of low results due to poor external validity and reproducibility of findings. Conversely, we observed a slight improvement in the quality of recommendations reported by CPG published after 2018, which may be due to greater use of the AGREE and GRADE instruments as a form of critical assessment of evidence and more careful elaboration of clinical guidelines.

Like Seow et al. [45] analysis of insomnia guidelines, we reported that the most productive regions of PAH guidelines were North America, Asia, and Europe, following the global geographical distribution of researchers, available technologies, science funding, and international collaboration [46]. However, although documents from Europe and some from North America scored slightly better in the AGREE II and AGREE REX tools, no significant differences were found in the overall quality of these guidelines. Likewise, guidelines’ scores did not differ according to the type of developer (society or government), which is consistent with the findings by De Melo et al. [41], who reported higher quality documents from professional societies and government institutions compared to independent researchers/universities. This may occur due to the different financial and human resources available in these organizations, especially considering CPG development is a time-consuming and costly process, as well as their scope and purposes.

Regarding the apparent lack of relationship between recommendations on PAH target therapy and AGREE II and AGREE REX quality, it is important to consider that there is no conclusive evidence comparing monotherapy and combination therapy or classes and drugs within classes. Therefore, the expert’s opinion is decisive on this matter, and thus, the influence of one guideline on another is notable, regardless of its quality.

Overall, future studies should prioritize methodological rigor (e.g., by means of systematic evaluations of existing guidelines to highlight areas where evidence is scarce or of poor quality), stakeholders’ engagement (e.g., qualitative research with peers, patients, caregivers, healthcare providers, and policymakers on their values and preferences can provide valuable insights regarding the real-world needs and challenges in PAH management), and clinical applicability and implementability (e.g., quality improvement initiatives including assessing barriers and facilitators aimed at testing the cost-effectiveness, feasibility, and adherence of treatment recommendations in clinical settings) [47, 48]. Moreover, although creating and updating high-quality guidelines every 2–5 years, as recommended, may be a costly process, especially for a small national professional society or governmental body, further strategies, including international consortiums, gathering external funding, and adaptations of high-quality published documents, are considered efficient. The use of evidence-to-decision frameworks can also provide better documents with more credible and transparent recommendations [1, 48].

Our review has some limitations. We only included freely available guidelines published after 2012, the year of the last update of the PAH treatment algorithm, which addressed pharmacological treatments; thus, results and conclusions cannot be generalized to other conditions. Nonetheless, a systematic and replicable methodology was performed following international recommendations and using four databases. Given the high heterogeneity among guidelines (i.e., year of publication, countries, target populations, and different methods to assess the level/certainty of evidence and propose recommendations), comparisons between guideline content should be avoided [27]. AGREE tools rely on inherent subjectivity when scoring their domains and may not fully account for the level of evidence attributed to recommendations, which can be subject to debate (e.g., perceptions of the tools and their measurement properties). Nonetheless, these instruments provide important frameworks to constantly evaluate and critically assess clinical practice guidelines. Although overall agreement across reviewers was considered substantial in our study, the evaluation of some items was arbitrary, leading to low consensus rates; however, this may reflect the opinions of a multidisciplinary team with different clinical experiences.

## Conclusions

Although some slight improvements in the report of recommendations from CPG on PAH pharmacological treatment occurred in the past years, methodological weaknesses are still common in the documents addressing this rare disease, especially regarding scientific rigor, values and preferences, and implementability. Thus, particular attention should be given to stakeholders’ perspectives and clinical applicability for developing future guidelines in this field.

## Supplementary Information

Below is the link to the electronic supplementary material.

Supplementary file1 (DOCX 461 KB)

## Data Availability

All data generated or analyzed during this study are included in this published article (and its supplementary information files). Further information are available from the corresponding author on reasonable request.
