# Supplementary material for: Methodological Quality of Pulmonary Arterial Hypertension Treatment Evidence-Based Guidelines: A Systematic Review Using the AGREE II and AGREE REX Tools
Source: Cardiovasc Drugs Ther. 2024 Jul 9;39(6):1353–63. doi: 10.1007/s10557-024-07605-w (PMC12717148; doi:10.1007/s10557-024-07605-w)
Supplement: Supplementary file 1 — Supplementary file1 (DOCX 461 KB) [file 10557_2024_7605_MOESM1_ESM.docx]

**Methodological quality of pulmonary arterial hypertension treatment evidence-based guidelines: a systematic review using the AGREE II and AGREE REX tools**

Ana Paula Oliveira Vilela, MsC. Pharmaceutical Assistance Postgraduate Program, Federal University of Paraná, Curitiba, Brazil (paulavili@hotmail.com)- ORCID ID 0009-0009-5162-313X

Flávia Deffert, PhD. Pharmaceutical Sciences Postgraduate Program, Universidade Federal do Paraná, Curitiba, Brazil (flaviadeffert@gmail.com) - ORCID ID 0000-0002-2188-9967

Rosa Camila Lucchetta, PhD. Health Technology Assessment Unit, Oswaldo Cruz German Hospital, São Paulo, Brazil (rc.lucch@yahoo.com.br – ORCID ID 0000-0002-4004-1320

Yara Maria da Silva Pires, MsC. Pharmaceutical Sciences Postgraduate Program, Federal University of Paraná, Curitiba, Brazil (yarapires@ufpr.br) – ORCID ID 0000-0003-1602-6566

Felipe Fernando Mainka, MsC. Pharmaceutical Sciences Postgraduate Program, Federal University of Paraná, Curitiba, Brazil (felipemainka@hotmail.com) – ORCID ID 0000-0002-8464-3286

Fernanda S. Tonin*, PhD, Professor. H&TRC - Health & Technology Research Center, ESTeSL - Escola Superior de Tecnologia da Saúde, Instituto Politécnico de Lisboa, Lisbon, Portugal; Pharmaceutical Sciences Postgraduate Program, Federal University of Paraná, Curitiba, Brazil (fernanda.tonin@estesl.ipl.pt) – ORCID ID 0000-0003-4262-8608

Roberto Pontarolo, PhD, Professor Department of Pharmacy, Federal University of Paraná, Curitiba, Brazil (pontarolo@ufpr.br) - ORCID ID 0000-0002-7049-4363

**Corresponding author*:**

**Fernanda S. Tonin:** H&TRC-Health & Technology Research Center, ESTeSL-Escola Superior de Tecnologia da Saúde, Instituto Politécnico de Lisboa. Av. Dom João II Lote 4.69 01, 1990-096 Lisbon, Portugal. Email: fernanda.tonin@estesl.ipl.pt

**Supplementary Information**

**Table 1 - Search strategy for the systematic review**

**Table 2 - Summary of the excluded studies after full-text appraisal and reasons exclusion**

**Table 3 - Overall characteristics of the 31 included guidelines**

**Table 4 - AGREE II and AGREE REX scores over time**

**Table 5 - Spearman correlation crossing domains and overall assessment score of AGREE II and AGREE REX tools**

**Table 6 - Association between AGREE II and AGREE REX scores according to type of developers**

**Table 7 - Association between AGREE II and AGREE REX scores and guidelines’ geographic origin**

**Table 8 - Recommendations from pulmonary arterial hypertension guidelines**

**Figure 1 - AGREE II and AGREE-REX results according to guidelines' geographic region**

**Figure 2 - Summary of recommendations about class of PAH target therapy, according AGREE II and AGREE REX scores.**

**Table 1**. Search strategy for the systematic review

| **Search** | **Search strategy** |
| --- | --- |
| PubMed  n= 2963 | Guide*[TIAB] OR recommendation*[TIAB] OR consensus [TIAB] OR Consensus [MH] OR “Health Planning Guidelines” [MH] OR “Guidelines as Topic” [MH] OR “Practice Guideline” [PT] OR Guideline [PT]  AND  “Pulmonary arterial hypertension” [TIAB] OR “pulmonary artery hypertension” [TIAB] OR “pulmonary hypertension” [TIAB] OR “Hypertension, Pulmonary” [MH] |
| Cochrane  n= 136 | (Guide OR Consensus OR recommendation *)  AND  **"Pulmonary Arterial Hypertension" OR” pulmonary** artery hypertension" **OR** “pulmonary hypertension**”"** |
| Embase  n= 1592 | Guideline**:ab,ti** OR Consensus**:ab,ti** OR recommendations**:ab,ti**  AND  **“pulmonary arterial hypertension”: ab,ti OR “**pulmonary artery hypertension”:ab,ti OR **“**pulmonary hypertension**”:ab,ti** |
| Tripdatabase  n= 2349 | hypertension AND pulmonary OR pulmonary AND arter* OR pulmonary arterial hypertension OR hypertension, pulmonary OR pulmonary Artery  AND  Guideline OR Consensus OR protocol OR guide OR recommendation |
| Searches were limited (i.e., by filters) to registers published after 2012 | |

**Table 2**. Summary of the excluded studies after full-text appraisal and reasons exclusion

| **Authors, year** | **Title** | **Reason for exclusion** |
| --- | --- | --- |
| Sallmon et al., 2019 | Recommendations from the Association for European Pediatric and Congenital Cardiology for training in pulmonary hypertension | Wrong publication |
| Vazquez ZGS; Klinger JR, 2020. | Guidelines for the Treatment of Pulmonary Arterial Hypertension | Review |
| Yaghi S; Novikov A; Trandafirescu T, 2020. | Clinical update on pulmonary hypertension. | Review |
| Wacker J; Weintraub R; Beghetti M, 2019. | An update on current and emerging treatments for pulmonary arterial hypertension in childhood and adolescence | Review |
| Mamedov MN, 2016. | A Review of Recent European Clinical Guidelines: What Useful for the Practitioner? | Review |
| Mejía Chew CR; Alcolea Batres S; Ríos Blanco JJ, 2016. | Update in pulmonary arterial hypertension | Review |
| Orfanos SE; Giannakoulas G, 2022. | Pulmonary Hypertension: Current Diagnosis, Approach and Treatment at the Dawn of the New European Guidelines. | Wrong publication |
| Working Group on Pulmonary Vascular Diseases of Chinese Society of Cardiology of Chinese Medical Association, 2018. | Chinese guidelines for the diagnosis and treatment of pulmonary hypertension 2018 | Article in Mandarin (Chinese) |
| Delgado V; Pascual D, 2022. | Comments to the 2022 ESC/ERS guidelines for the diagnosis and treatment of pulmonary hypertension. | Wrong publication |
| Working Group hypertension Diseases World Health Organisation, 2018. | Guideline for the pharmacological treatment of hypertension in adults: web annex A: summary of evidence | clinical guideline for another disease |
| Sirajuddin et al., 2022 | ACR Appropriateness Criteria® Suspected Pulmonary Hypertension: 2022 Update | Wrong publication |
| Karmakar, S.; Paul, M.; Sengupta, S.; Bhattacharyya, P, 2022. | Treating PH in DPLD in real world: A consensus approach and results | Wrong publication |
| Foris, V.; Kovacs, G.; Olschewski, H, 2021. | Update on pulmonary hypertension focusing on lung diseases | Article in German |
| Hasan, B et al., 2020. | Challenges and Special Aspects of Pulmonary Hypertension in Middle- to Low-Income Regions: JACC State-of-the-Art Review | Wrong publication |
| Humpl, T.,2019. | Recommendations for action according to the S2k guidelines on Pulmonary arterial hypertension (PAH) in childhood and adolescence | Article in German |
| Rosenkranz, S et al., 2016. | Pulmonary Hypertension: Cologne Consensus Conference 2016 | Article in German |
| Leuchte, H.H et al., 2016. | Risk stratification and follow-up assessment of patients with pulmonary arterial hypertension: Recommendations of the Cologne Consensus Conference 2016 | Article in German |
| Grünig, E et al., 2016 | General and supportive therapy of pulmonary arterial hypertension | Article in German |
| Hoeper, M.M et al., 2016. | Targeted therapy of pulmonary arterial hypertension: Recommendations of the Cologne Consensus Conference 2016 | Article in German |
| Olschewski, H et al., 2016. | Pulmonary hypertension due to chronic lung disease: Recommendations of the Cologne Consensus Conference 2016 | Article in German |
| Wilkens, H et al., 2016. | Chronic thromboembolic pulmonary hypertension: Recommendations of the Cologne Consensus Conference 2016 | Article in German |
| Qian, J.; Li, M.; Wang, Y.; Zeng, X, 2016. | Diagnosis, evaluation and management of SLE-associated pulmonary arterial hypertension in Chinese adults: Recommendations from Chinese SLE Treatment and Research Group | Wrong publication |
| Latus, H et al., 2016. | Cardiac MR and CT imaging in children with suspected or confirmed pulmonary hypertension/pulmonary hypertensive vascular disease. Expert consensus statement on the diagnosis and treatment of paediatric pulmonary hypertension. The European Paediatric Pulmonary Vascular Disease Network, endorsed by ISHLT and DGPK | Wrong publication |
| Pattathu, J et al., 2016. | Genetic testing and blood biomarkers in paediatric pulmonary hypertension. Expert consensus statement on the diagnosis and treatment of paediatric pulmonary hypertension. The European Paediatric Pulmonary Vascular Disease Network, endorsed by ISHLT and DGPK | Wrong publication |
| Olschewski, H.; Kovacs, G, 2015. | ESC guidelines 2015 on pulmonary hypertension | Article in German |
| Y Pei, W Tang, 2015. | Expert consensus for the diagnosis and management of pediatric pulmonary hypertension | Article in Mandarin (Chinese) |
| Hassell, K.L et al., 2014. | Practice guideline for pulmonary hypertension in sickle cell: Direct evidence needed before universal adoption | Wrong publication |
| Fang, J.C., 2013. | Erratum: WHO Pulmonary Hypertension Group 2: Pulmonary hypertension due to left heart disease in the adult. A consensus statement of the PH council of the ISHLT JHLT 2012 | Wrong publication |
| Sitbon, O et al., 2012. | Treat-to-target approach in pulmonary arterial hypertension: A consensus-based proposal | Wrong publication |
| SN Avdeev - Terapevticheskii arkhiv, 2012. | Current recommendations on diagnosis and treatment of pulmonary arterial hypertension | Article in Russian |
| Sockrider M., 2021. | Diagnosis and Treatment of Pulmonary Hypertension. | Wrong publication |
| Kovacs G, et al., 2016. | Clinical classification and initial diagnosis of pulmonary hypertension: recommendations of the Cologne Consensus Conference 2016 | Article in German |
| Sarybaev AS, et al., 2017. | Diagnosis and treatment of pulmonary hypertension: from the point of view of 2017 | Article in German |
| Hambly N; Alawfi F; Mehta S, 2016. | Pulmonary hypertension: diagnostic approach and optimal management | Wrong publication |
| Rosenkranz S et al., 2016. | Pulmonary hypertension associated with left heart disease: recommendations of the Cologne Consensus Conference 2016 | Article in German |
| Sommer N et al., 2017 | Update pulmonary arterial hypertension: Definitions, diagnosis, therapy | Article in German |
| Opitz C et al., 2016. | ESC guidelines 2015 pulmonary hypertension: diagnosis and treatment | Article in German |
| Rådegran G, 2021. | National consensus created regarding PAH and CTEPH care | Article in Swedish |
| Correale M et al., 2017. | European guidelines on pulmonary hypertension | Article in Italian |
| Jerjes-Sánchez C et al., 2022 | Riociguat in the Treatment of Pulmonary Arterial Hypertension | Wrong publication |
| Dardi F, 2021. | A pragmatic approach to risk assessment in pulmonary arterial hypertension using the 2015 European Society of Cardiology/European Respiratory Society guidelines | Wrong publication |
| ler SS et al., 2022. | Building a dedicated pediatric pulmonary hypertension program: A consensus statement from the pediatric pulmonary hypertension network | Wrong publication |
| Barnett CF; De Marco T; Galiè N, 2022. | Where we came from and where we are going: A perspective on the practice changing recommendations from the 2022 ESC/ERS pulmonary hypertension guidelines | Wrong publication |
| Canadian Agency for Drugs and Technologies in Health, 2016. | CADTH Canadian Drug Expert Committee Final Recommendation Selexipag | Wrong publication |
| Galiè N et al., 2016. | 2015 ESC/ERS Guidelines for the Diagnosis and Treatment of Pulmonary Hypertension. | The document is not a clinical guideline |
| Kristin E Schwab; **Nader Kamangar, 2020.** | Pulmonary Arterial Hypertension | Review |
| Ronald J Oudiz et al., 2020. | Idiopathic Pulmonary Arterial Hypertension | Review |
| Poothirikovil Venugopalan et al., 2020. | High Altitude Pulmonary Hypertension | Review |
| Paresh Chandra Giri et al., 2020. | Group 4 Pulmonary Hypertension | Review |
| Nikhil Barot, 2020. | Group 2 Pulmonary Hypertension | Review |

T**able 3**. Overall characteristics of the 31 included guidelines

| Guidelines | CPG Title | Geographic origin | Type of organization | Target users | Occupation of the listed authors |
| --- | --- | --- | --- | --- | --- |
| AHA-ATS 2015 | Executive summary of the American Heart Association and American thoracic society joint guidelines for pediatric pulmonary hypertension. | North America | professional society | Health Professionals | Physicians |
| CCC 2018 | Chronic thromboembolic pulmonary hypertension (CTEPH): Updated Recommendations from the Cologne Consensus Conference 2018. | Europe | professional society | Health Professionals | Physicians |
| EPPVDN 2016 | Pulmonary hypertension in the intensive care unit. Expert consensus statement on the diagnosis and treatment of pediatric pulmonary hypertension. The European Pediatric Pulmonary Vascular Disease Network, endorsed by ISHLT and DGPK. | Europe | professional society | Health Professionals | Physicians |
| EPPVDN 2019 | 2019 updated consensus statement on the diagnosis and treatment of pediatric pulmonary hypertension: The European Pediatric Pulmonary Vascular Disease Network (EPPVDN), endorsed by AEPC, ESPR and ISHLT. | Europe | professional society | Health Professionals | Physicians |
| ESC ERS 2015 | Guidelines for the diagnosis and treatment of pulmonary hypertension: The Joint Task Force for the Diagnosis and Treatment of Pulmonary Hypertension of the European Society of Cardiology (ESC) and the European Respiratory Society (ERS): Endorsed by: Association for European Paediatric and Congenital Cardiology (AEPC), International Society for Heart and Lung Transplantation (ISHLT). | Europe | professional society | Health Professionals | Physicians |
| ESC ERS 2022 | Guidelines for the diagnosis and treatment of pulmonary hypertension: Developed by the task force for the diagnosis and treatment of pulmonary hypertension of the European Society of Cardiology (ESC) and the European Respiratory Society (ERS) | Europe | professional society | Health Professionals | Physicians |

| JCS-JPCPHS 2019 | Guidelines for the Treatment of Pulmonary Hypertension. | Asia - Japan | professional society | Health Professionals | Physicians |
| --- | --- | --- | --- | --- | --- |
| PCICS 2014 | Pediatric cardiac intensive care society 2014 consensus statement: Pharmacotherapies in cardiac critical care pulmonary hypertension. | North America | professional society | Health Professionals | Physicians and pharmacists |
| SAUDI 2014 | Guidelines on the diagnosis and treatment of pulmonary hypertension: 2014 updates. | Asia - Saudi Arabia | professional society | Health Professionals | Physicians |
| TSOC 2014 | Guidelines of Taiwan Society of Cardiology (TSOC) for the Management of Pulmonary Arterial Hypertension. | Asia - China | professional society | Health Professionals | Physicians |
| TSOC 2018 | Updated guideline focused on diagnosis and treatment of pulmonary arterial hypertension. | Asia - China | professional society | Health Professionals | Physicians and physiotherapist |
| 6^th^ SYMPOSIUM 2019 | Pediatric pulmonary arterial hypertension: updates on definition, classification, diagnoses and management. | North America | professional society | Health Professionals | Physicians |
| ATS 2014 | An official American Thoracic Society clinical practice guideline: diagnosis, risk stratification, and management of pulmonary hypertension of sickle cell disease. | North America | professional society | Health Professionals | Physicians |
| SBPT 2022 | Brazilian Thoracic Society recommendations for the diagnosis and treatment of chronic thromboembolic pulmonary hypertension. | South America - Brazil | professional society | Health Professionals | Physicians |
| CCS-CTS 2020 | Canadian Cardiovascular Society/Canadian Thoracic Society Position Statement on Pulmonary Hypertension. | North America | professional society | Health Professionals | Physicians |
| CHEST 2014 | Pharmacologic therapy for pulmonary arterial hypertension in adults: CHEST guideline and expert panel report. | North America | professional society | Health Professionals | Physicians |
| CHEST 2019 | Therapy for Pulmonary Arterial Hypertension in Adults: Update of the CHEST Guideline and Expert Panel Report. | North America | professional society | Health Professionals | Physicians |
| CHINA 2020 | Chinese Expert-based Consensus on the Diagnosis and Treatment of Connective Tissue Disease Associated Pulmonary Arterial Hypertension. | Asia - China | governmental | Health Professionals | Physicians |
| CSC 2019 | Update of the Colombian Consensus on Heart Failure with Reduced Ejection Fraction: Chapter on Heart Failure, Heart Transplantation and Pulmonary Hypertension of the Colombian Society of Cardiology and Cardiovascular Surgery. | South America - Colombia | professional society | Health Professionals | Physicians |
| USA 2016 | New Therapeutic Paradigms and Guidelines in the Management of Pulmonary Arterial Hypertension. | North America | professional society | Health Professionals | Physicians and pharmacists |
| PAH-MS 2014 | Clinical Protocol and Therapeutic Guidelines for Pulmonary Arterial Hypertension. | South America - Brazil | governmental | Health Professionals | Physicians |
| HAT 2021 | Executive summary thai pulmonary hypertension guidelines. | Asia - Thailand | professional society | Health Professionals | Physicians |
| KSC-KATRD 2020 | Guideline for the Diagnosis and Treatment of Pulmonary Hypertension: Executive Summary. | Asia - Korea | professional society | Health Professionals | Physicians |
| PROSTACYCLINE 2020 | Results of an Expert Consensus Survey on the Treatment of Pulmonary Arterial Hypertension with Oral Prostacyclin Pathway Agents. | North America | governmental | Health Professionals | Physicians |
| SA HEART 2015 | Management of pulmonary hypertension | Africa - South Africa | professional society | Health Professionals | Physicians |
| SEPAR 2018 | Guidelines on the Diagnosis and Treatment of Pulmonary Hypertension: Summary of Recommendations. | Europe | professional society | Health Professionals | Physicians |
| C.TREPROSTINIL 2020 | An Expert Panel Delphi Consensus Statement on Patient Selection and Management for Transitioning Between Oral and Inhaled Treprostinil. | North America | professional society | Health Professionals | Physicians |
| C.RIOCIGUAT 2022 | Delphi consensus recommendation for optimization of pulmonary hypertension therapy focusing on switching from a phosphodiesterase 5 inhibitor to riociguat. | North America | professional society | Health Professionals | Physicians |
| C.MANAGEMENT 2021 | Establishing expert consensus for the optimal approach to holistic risk-management in pulmonary arterial hypertension: a Delphi process and narrative review. | Europe | professional society | Health Professionals | Physicians, pharmacists, and nurses |
| C.CARE 2022 | An expert panel Delphi consensus statement on the use of palliative care in the management of patients with pulmonary arterial hypertension | North America | professional society | Health Professionals | Physicians, pharmacists, and nurses |
| WHO 2012 | World Health Organization Pulmonary Hypertension group 2: pulmonary hypertension due to left heart disease in the adult--a summary statement from the Pulmonary Hypertension Council of the International Society for Heart and Lung Transplantation. | North America | professional society | Health Professionals | Physicians |

**Table 4**. AGREE II and AGREE REX scores over time

| **Score** | **Before 2018**  **(n=16)** | **From 2018**  **(n=15)** | **Mann-Whitney**  **U-value** | **Monte Carlo**  **p-value (95%CI)** |
| --- | --- | --- | --- | --- |
| **AII** | 63.50 (24.25) | 63.0 (18.0) | 107.5 | 0.621 (0.441:0.784) |
| **AII D1** | 92.50 (13.0) | 96.0 (14.0) | 113.0 | 0.780 (0.588:0.896) |
| **AII D2** | 56.0 (31.25) | 68 (37.0) | 84.5 | 0.160 (0.00:0.201) |
| **AII D3** | 31.5 (26.75) | 32.0 (35.0) | 107.5 | 0.621 (0.441:0.784) |
| **AII D4** | 99.0 (9.5) | 99.0 (7.0) | 115.0 | 0.839 (0.667:0.946) |
| **AII D5** | 46.0 (32.50) | 33.0 (33.0) | 87.5 | 0.199(0.054:0.333) |
| **AII D6** | 78.0 (58.25) | 83.0 (36.0) | 110.0 | 0.753 (0.692:0.989) |
| **AR** | 43.5 (23.0) | 56.0 (21.0) | 59.0 | 0.016 (0.00:0.094) |
| **AR D1** | 73.5 (22.5) | 76.0 (19.0) | 101.0 | 0.452 (0.276:0.627) |
| **AR D2** | 17.0 (21.75) | 44.0(22.00) | 58.0 | 0.014 (0.00:0.092) |
| **AR D3** | 42.5 (24.0) | 60.0 (25.0) | 65.0 | 0.029 (0.00:0.201) |
| AGREE II domains: (AII) Overall assessment; (AII D1) Domain 1 – Scope and Purpose; (AII D2) Domain 2 – Stakeholder Involvement; (AII D3) Domain 3 – Rigour of Development; (AII D4) Domain 4 – Clarity of Presentation; (AII D5) Domain 5 – Applicability; (AII D6) Domain 6 – Editorial Independence.  AGREE REX domains: (AR) Overall assessment; (AR D1) Domain 1 – Clinical Applicability; (AR D2) Domain 2 – Values and Preferences; (AR D3) Domain 3 – Implementability. | | | | |

**Table 5.** Spearman correlation crossing domains and overall assessment score of AGREE II and AGREE REX tools

|  | **AII D1** | **AII D2** | **AII D3** | **AII D4** | **AII D5** | **AII D6** | **AR** | **AR D1** | **AR D2** | **AR D3** |
| --- | --- | --- | --- | --- | --- | --- | --- | --- | --- | --- |
| **AII** | 0.391 | 0.630 | 0.813 | 0.516 | 0.745 | 0.649 | 0.722 | 0.654 | 0.604 | 0.547 |
| **AII D1** | ns | ns | 0.397 | ns | ns | ns | 0.422 | ns | 0.522 | ns |
| **AII D2** | ns | ns | 0.631 | 0.423 | 0.405 | ns | 0.629 | 0.586 | 0.531 | 0.476 |
| **AII D3** | 0.397 | 0.631 | ns | 0.410 | 0.649 | ns | 0.771 | 0.719 | 0.616 | 0.619 |
| **AII D4** | ns | 0.423 | 0.410 | ns | 0.593 | ns | 0.450 | 0.490 | ns | 0.392 |
| **AII D5** | ns | 0.405 | 0.649 | 0.593 | ns | 0.355 | 0.497 | 0.612 | ns | 0.415 |
| **AII D6** | ns | ns | ns | ns | 0.355 | ns | ns | ns | ns | ns |
| **AR** |  |  |  |  |  |  | ns | 0.722 | 0.837 | 0.880 |
| **AR D1** |  |  |  |  |  |  | 0.722 | ns | 0.379 | 0.565 |
| **AR D2** |  |  |  |  |  |  | 0.837 | 0.379 | ns | 0.596 |
| **Note**: AGREE II domains: (AII) Overall assessment; (AII D1) Domain 1 – Scope and Purpose; (AII D2) Domain 2 – Stakeholder Involvement; (AII D3) Domain 3 – Rigour of Development; (AII D4) Domain 4 – Clarity of Presentation; (AII D5) Domain 5 – Applicability; (AII D6) Domain 6 – Editorial Independence; AGREE REX domains: (AR) Overall assessment; (AR D1) Domain 1 – Clinical Applicability; (AR D2) Domain 2 – Values and Preferences; (AR D3) Domain 3 – Implementability. ns: not significant. | | | | | | | | | | |

**Table 6.** Association between AGREE II and AGREE REX scores according to type of developers

| **Domains** | **Society**  **(n=28)** | **Government**  **(n=3)** | **Mann-Whitney**  **U-value** | **Monte Carlo**  **p-valor (95%IC)** |
| --- | --- | --- | --- | --- |
| **AII** | 64 (24.0) | 61.0 (9.5) | 43.0 | 0.516 (0.308:0.660) |
| **AII D1** | 93.0 (14) | 95.5 (12.8) | 44.5 | 0,572 (0.513:0.842) |
| **AII D2** | 63.0 (38.0) | 64.0 (13.6) | 53.0 | 0,953 (0.908:1.00) |
| **AII D3** | 32.0 (25.0) | 35.0 (38.8) | 52.0 | 0.908 (0.906:1.00) |
| **AII D4** | 99.0 (8.0) | 96.5 (8.8) | 52.5 | 0.928 (0.906:1.00) |
| **AII D5** | 42.0 (35.0) | 26.5 (31.3) | 31.5 | 0.185 (0.054:0.333) |
| **AII D6** | 83.0 (36.0) | 63.5 (68.3) | 42.0 | 0.479 (0.246:0.593) |
| **AR** | 51.0 (21.0) | 54.0 (22.5) | 51.0 | 0.860 (0.667:0.946) |
| **AR D1** | 75.0 (19.0) | 72.0 (22.3) | 50.5 | 0.836 (0.709:0.968) |
| **AR D2** | 35.0 (34.0) | 29.5 (25.5) | 45.5 | 0.616 (0.477:0.814) |
| **AR D3** | 46.0 (28.0) | 57.50 (23.5) | 36.0 | 0.288 (0.131:0.450) |
| AGREE II domains: (AII) Overall assessment; (AII D1) Domain 1 – Scope and Purpose; (AII D2) Domain 2 – Stakeholder Involvement; (AII D3) Domain 3 – Rigour of Development; (AII D4) Domain 4 – Clarity of Presentation; (AII D5) Domain 5 – Applicability; (AII D6) Domain 6 – Editorial Independence.  AGREE REX domains: (AR) Overall assessment; (AR D1) Domain 1 – Clinical Applicability; (AR D2) Domain 2 – Values and Preferences; (AR D3) Domain 3 – Implementability. | | | | |

**Table 7.** Association between AGREE II and AGREE REX scores and guidelines’ geographic origin

| **Domain**  **Region (n)** | **Mediana (IR)** | **Kruskal Wallis** | **Monte Carlo (IC 95%)** |
| --- | --- | --- | --- |
| **AII**  Asia (7)  Europe (6)  North America (14)  South America (3)  Africa* (1) | 64.0 (16.0)  76.0 (15,3)  62.0(27.0)  58.0 (-)  - | X² (4) =8.354; p=0.79 | 0.53 (0.048:0.057) |
| **AII D1**  Asia (7)  Europe (6)  North America (14)  South America (3)  Africa* (1) | 96.0 (16.0)  93.0 (9.3)  95.0(16.8)  89.0 (-)  - | X² (4) =0.141; p=0.998 | 0.999 (0.998:0.999) |
| **AII D2**  Asia (7)  Europe (6)  North America (14)  South America (3)  Africa* (1) | 57.0 (21.0)  79.0 (44.3)  64.0 (39.8)  56.0 (-)  - | X² (4) =2.724; p=0.605 | 0.660 (0.651:0.669) |
| **AII D3**  Asia (7)  Europe (6)  North America (14)  South America (3)  Africa* (1) | 27.0 (11.0)  51.0 (35.0)  32.5 (46.3)  26.0 (-)  - | X² (4) =7.635; p=0.106 | 0.081 (0.076:0.087) |
| **AII D4**  Asia (7)  Europe (6)  North America (14)  South America (3)  Africa* (1) | 99.0 (3.0)  99.0 (8.5)  97.0 (11.8)  99.0(-)  - | X² (4) =4.520; p=0.340 | 0.354 (0.345:0.363) |
| **AII D5**  Asia (7)  Europe (6)  North America (14)  South America (3)  Africa* (1) | 48.0 (25.0)  56.5 (25.0)  31.0 (37.3)  33.0 (-)  - | X² (4) =8.039; p=0.090 | 0.065 (0.060:0.070) |
| **AII D6**  Asia (7)  Europe (6)  North America (14)  South America (3)  Africa* (1) | 83.0 (71.0)  85.5 (17.5)  77.0 (34.5)  50.0 (-)  - | X² (4) =4.937; p=0.294 | 0.291(0.282:0.299) |
| **RA**  Asia (7)  Europe (6)  North America (14)  South America (3)  Africa* (1) | 47.0 (21.0)  56.0 (27.5)  53.0 (21.5)  52.0 (-)  - | X² (4) =3.222; p=0.521 | 0.569(0.559-0.578) |
| **AR D1**  Asia (7)  Europe (6)  North America (14)  South America (3)  Africa* (1) | 72.0 (18.0)  77.0 (13.3)  64.5 (25.5)  83.0 (-)  - | X² (4) =6.229; p=0.183 | 0.165(0.158:0.172) |
| **AR D2**  Asia (7)  Europe (6)  North America (14)  South America (3)  Africa* (1) | 28.0 (28.0)  31.5 (35.8)  43.5 (41.3)  25.0 (-)  - | X² (4) =2.092; p=0.719 | 0.776(0.768-0.785) |
| **AR D3**  Asia (7)  Europe (6)  North America (14)  South America (3)  Africa* (1) | 46.0 (33.0)  58.0 (38.0)  46.0 (25.5)  60.0 (-)  - | X² (4) =3.041; p=0.551 | 0.601(0.591:0.610) |
| AGREE II domains: (AII) Overall assessment; (AII D1) Domain 1 – Scope and Purpose; (AII D2) Domain 2 – Stakeholder Involvement; (AII D3) Domain 3 – Rigour of Development; (AII D4) Domain 4 – Clarity of Presentation; (AII D5) Domain 5 – Applicability; (AII D6) Domain 6 – Editorial Independence.  AGREE REX domains: (AR) Overall assessment; (AR D1) Domain 1 – Clinical Applicability; (AR D2) Domain 2 – Values and Preferences; (AR D3) Domain3 – Implementability.  *it has been omitted | | | |

**Table 8.** Recommendations from pulmonary arterial hypertension guidelines

| Guideline | AII | AR | Low risk | Intermediate risk | High risk |
| --- | --- | --- | --- | --- | --- |
| CHEST 2019 | 89 | 81 | Monotherapy (ERA, PDE5I, or GC) or dual combination (ERA+PDE5I) | Monotherapy (ERA, PDE5I, or GC) or dual combination (ERA+PDE5I) or triple therapy, including a prostanoid | Monotherapy (prostanoid) or dual combination (prostaoind and PDE5I or ERA) |
| ESC ERS 2022 | 91 | 71 | Dual therapy (ERA+PDE5I) | Dual therapy (ERA+PDE5I) | Triple therapy (prostanoid+PDE5I+ERA) |
| CHEST 2014 | 87 | 69 | Monotherapy (ERA, PDE5I, or GC) | Monotherapy (ERA, PDE5I, or GC) or dual therapy, including prostanoid | Monotherapy (prostanoid) or dual therapy (prostanoid+ERA) |
| HAT 2021 | 71 | 68 | Monotherapy (ERA, PDE5I, prostanoid, or GC) | Monotherapy or combination therapy (ERA, PDE5I, prostanoid, and/or GC) | Combination therapy (ERA, PDE5I, PCA, and/or GC) |
| EPPVDN 2019 * | 81 | 68 | Monotherapy (PDE5I, ERA, or prostanoid) or dual therapy | Monotherapy (PDE5I, ERA, or prostanoid) or dual therapy | Prostanoid or triple therapy (prostanoid+PDE5I+ERA) |
| KSC-KATRD 2020 | 76 | 65 | Monotherapy or combination therapy (PDE5I, GC, and/or prostanoid) | Monotherapy or combination therapy (PDE5I, GC, and/or prostanoid) | Monotherapy or combination therapy, including prostanoid |
| PROSTACYCLIN 2020 ¥ | 70 | 64 | Dual combination (ERA+PDE5I with or without prostanoid) | Dual combination (ERA+PDE5I with or without prostanoid) | Dual combination (ERA+PDE5I with or without prostanoid) |
| ATS 2014 £ | 87 | 63 | NR | NR | NR |
| C.TREPROSTINIL 2020 ¤ | 57 | 60 | NA | NA | NA |
| ESC ERS 2015 | 81 | 60 | Monotherapy or dual therapy | Monotherapy or dual therapy | Dual therapy, including prostanoid |
| PAH-MS 2014 € | 58 | 60 | NR | NR | NR |
| C.CARE 2022 ⁋ | 65 | 56 | NA | NA | NA |
| CCS-CTS 2020 | 59 | 55 | Monotherapy (ERA, PDE5I, or GC) | Dual therapy (ERA, PDE5I, and/or GC) | Combination therapy (ERA, PDE5I, and/or GC), including prostanoid |
| CSC 2019 ⁑ | 63 | 52 | NR | NR | NR |
| SEPAR 2018 | 71 | 52 | Monotherapy or combination therapy (ERA, PDE5I, and/or GC) | Monotherapy or combination therapy (ERA, PDE5I, and/or GC) | Monotherapy (prostanoid) or triple combination, including prostanoid |
| C.RIOCIGUAT 2022 † | 63 | 51 | NA | NA | NA |
| CHINA 2020 € | 62 | 48 | NA | NA | NA |
| JCS 2019 | 55 | 47 | Monotherapy or combination therapy (ERA, PDE5I, GC, and/or prostanoid) | Combination therapy (ERA, PDE5I, GC, and/or prostanoid) | Combination therapy (ERA, PDE5I, GC, and/or prostanoid) |
| TSOC 2018 | 64 | 46 | Combination therapy (ERA+PDE5I) | Combination therapy (ERA+PDE5I) | Combination therapy (ERA+PDE5I+prostanoid) |
| 6th SYMPOSIUM 2019 * | 61 | 45 | Monotherapy or combination therapy (ERA, PDE5I, GC, and/or prostanoid) | NR | Monotherapy or combination therapy (ERA, PDE5I, GC, and/or prostanoid) |
| C.MANAGEMENT 2021 | 58 | 44 | Monotherapy | Combination therapy | Combination therapy, including prostanoid |
| SAUDI 2014 | 67 | 44 | Monotherapy or combination therapy (ERA and/or PDE5I) | Combination therapy (ERA and/or PDE5I) | Combination therapy, including prostanoid |
| AHA-ATS 2015 | 84 | 43 | NR | NR | Prostanoid |
| CCC 2018 ‡ | 70 | 42 | NA | NA | NA |
| SBPT 2020 ‡ | 53 | 41 | NR | NR | NR |
| EPPVDN 2016 § | 63 | 39 | NA | NA | NA |
| USA 2016 | 60 | 38 | Monotherapy or dual combination (ERA+PDE5I) | Monotherapy or dual combination (ERA+PDE5I) | Monotherapy (prostanoid) or dual combination (ERA+PDE5I+prostanoid) |
| WHO 2012 € | 53 | 35 | NA | NA | NA |
| TSOC 2014 | 50 | 34 | Monotherapy (ERA, PDE5I or GC) | Combination therapy (ERA, PDE5I, prostanoid and/or GC) | Combination therapy (ERA, PDE5I, prostanoid and/or GC) |
| PCICS 2014 *€ | 48 | 32 | Monotherapy or combination therapy (PDE5I, GC, and/or prostanoid) | NR | NR |
| SA HEART 2015 | 44 | 23 | NR | NR | NR |

**Note**: * Paediatric PAH; ¥ Focus is the criteria for prostacyclin agonist use; £ Focus is PAH in sickle cell disease and no recommendation is made considering functional class or risk; ¤ Focus is on switching from inhaled to oral treprostinil; € recommendation does not consider risk or functional class; ⁋ Focus is on palliative treatment; ⁑ Focus is heart failure and PAH is treated as a comorbidity; † Focus is on the exchange of PDE5I for riociguat; ‡ Focus is on chronic thromboembolic pulmonary hypertension; § Focus is on intensive care of paediatric PAH; §§ Does not report any recommendations for PAH target therapy. AII: Overall assessment on AGREE II; AR: Overall assessment on AGREE REX; ERA: Endothelin receptor antagonist; GC: Guanylate cyclase agonist; NA: not applicable; NR: not reported; PDE5I: Phosphodiesterase type-5 inhibitor; PAH: pulmonary arterial hypertension.


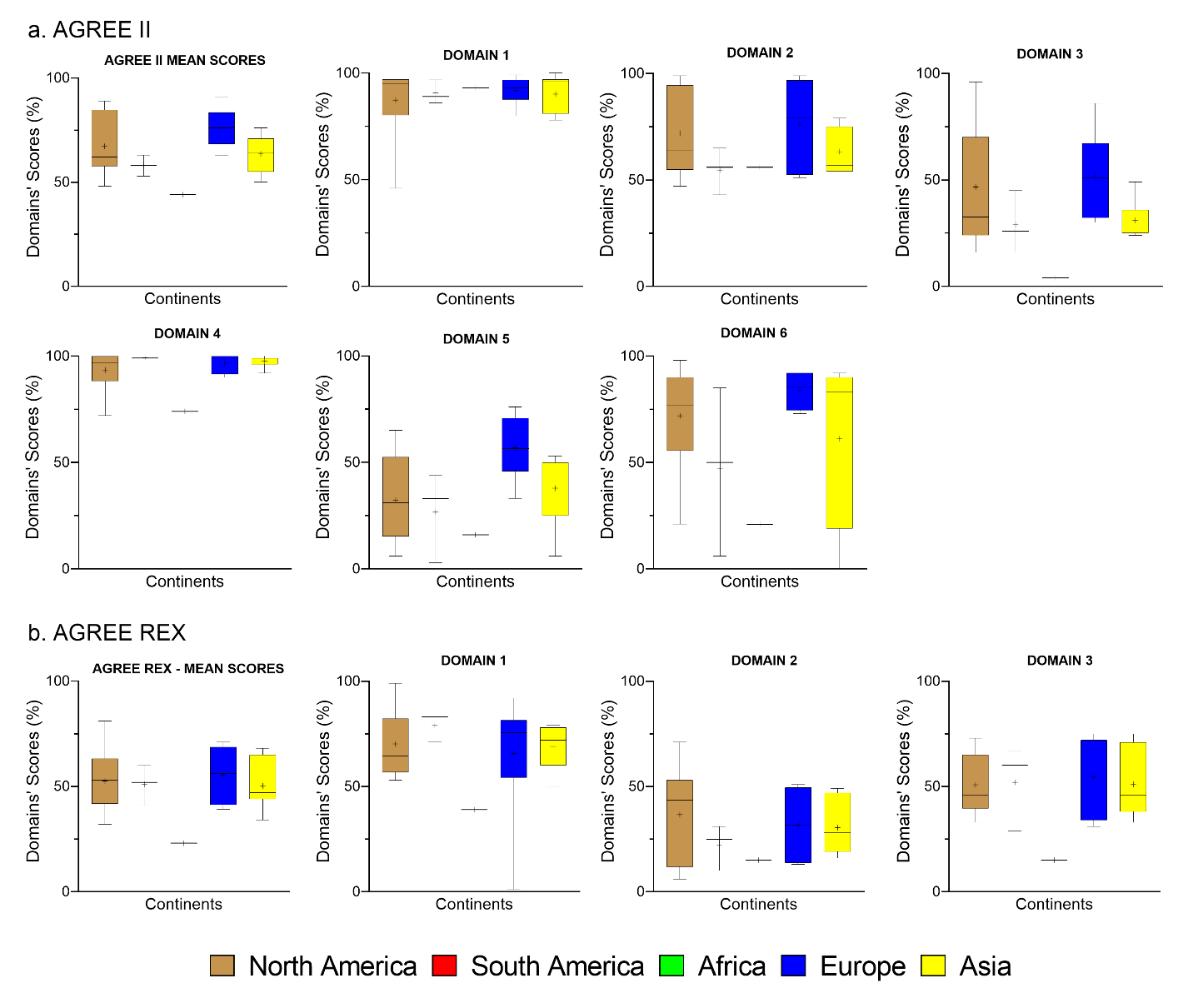


**Fig 1 AGREE II and AGREE-REX results according to guidelines' geographic region**

For all representations, the second and third boxplots are, respectively, South America and Africa. AGREE II domains: Domain 1 – Scope and Purpose; Domain 2 – Stakeholder Involvement; Domain 3 – Rigour of Development; Domain 4 – Clarity of Presentation; Domain 5 – Applicability; Domain 6 – Editorial Independence; AGREE REX domains: Domain 1 – Clinical Applicability; Domain 2 – Values and Preferences; Domain 3 – Implementability.


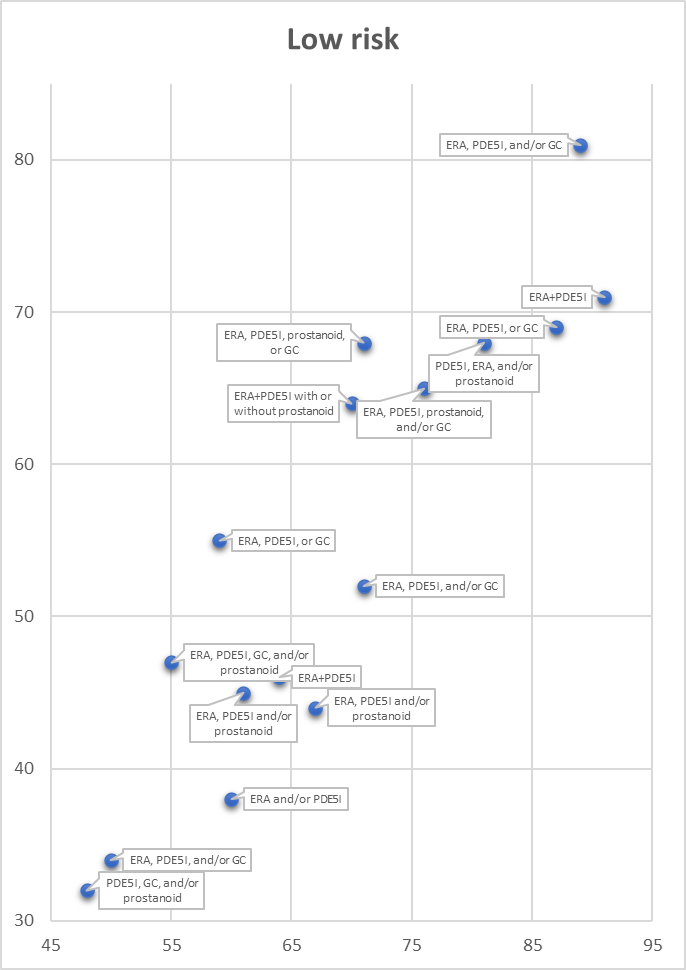
**
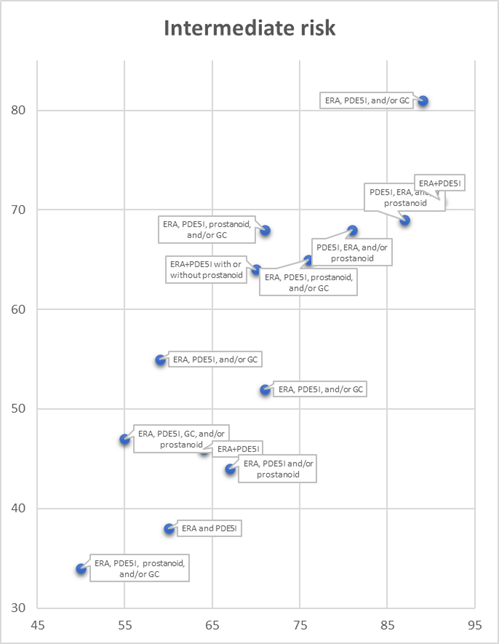
**
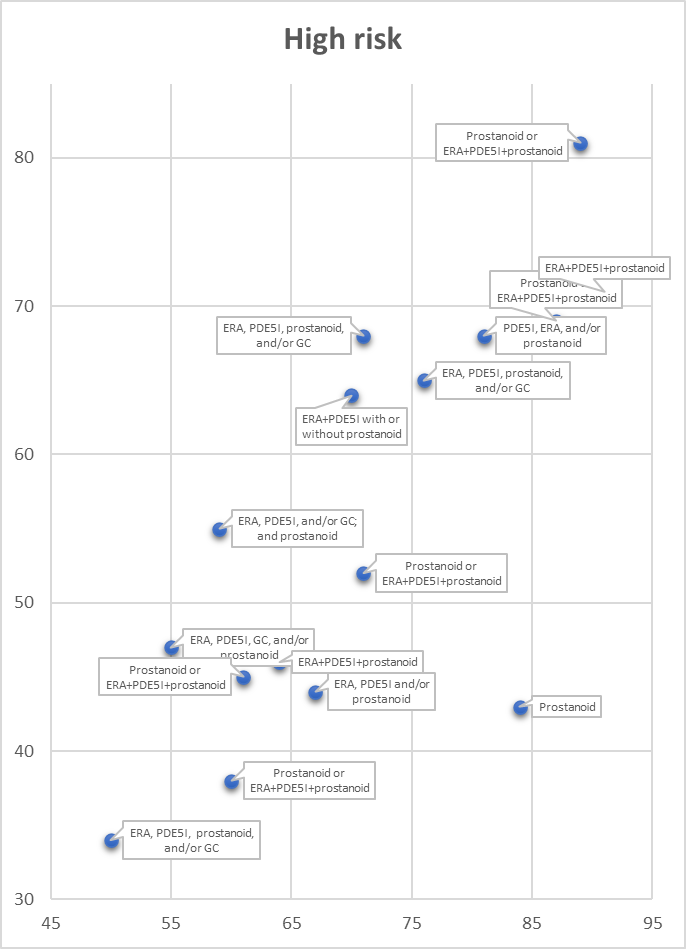


**Fig 2 Summary of recommendations about class of PAH target therapy, according AGREE II and AGREE REX scores**

Note: Only recommendations that considered the WHO functional class for risk categorization were considered. Guidelines that did not formulate recommendations according to risk categorization (not applicable) or that did not make the pharmacological class clear (not reported) were excluded from this analysis (see table 9). ERA: Endothelin receptor antagonist; GC: Guanylate cyclase agonist; PDE5I: Phosphodiesterase type-5 inhibitor.

**References of the included guidelines**

Hirani N, Brunner NW, Kapasi A et al. Canadian Cardiovascular Society/Canadian Thoracic Society Position Statement on Pulmonary Hypertension. The Canadian journal of cardiology. 2020;36(7):977-92. doi:10.1016/j.cjca.2019.11.041.

Klings ES, Machado RF, Barst RJ et al. An official American Thoracic Society clinical practice guideline: diagnosis, risk stratification, and management of pulmonary hypertension of sickle cell disease. American journal of respiratory and critical care medicine. 2014;189(6):727-40. doi:10.1164/rccm.201401-0065ST.

Taichman DB, Ornelas J, Chung L et al. Pharmacologic therapy for pulmonary arterial hypertension in adults: CHEST guideline and expert panel report. Chest. 2014;146(2):449-75. doi:10.1378/chest.14-0793.

Fang JC, DeMarco T, Givertz MM et al. World Health Organization Pulmonary Hypertension group 2: pulmonary hypertension due to left heart disease in the adult--a summary statement from the Pulmonary Hypertension Council of the International Society for Heart and Lung Transplantation. The Journal of heart and lung transplantation : the official publication of the International Society for Heart Transplantation. 2012;31(9):913-33. doi:10.1016/j.healun.2012.06.002.

Rosenzweig EB, Abman SH, Adatia I et al. Paediatric pulmonary arterial hypertension: updates on definition, classification, diagnostics and management. The European respiratory journal. 2019;53(1). doi:10.1183/13993003.01916-2018.

Rahaghi FF, Allen RP, Balasubramanian VP et al. An expert panel delphi consensus statement on patient selection and management for transitioning between oral and inhaled treprostinil. Pulmonary pharmacology & therapeutics. 2021;66:101979. doi:10.1016/j.pupt.2020.101979.

Rahaghi FF, Balasubramanian VP, Bourge RC et al. Delphi consensus recommendation for optimization of pulmonary hypertension therapy focusing on switching from a phosphodiesterase 5 inhibitor to riociguat. Pulmonary circulation. 2022;12(2):e12055. doi:10.1002/pul2.12055.

Church C, Gin-Sing W, Grady D et al. Establishing expert consensus for the optimal approach to holistic risk-management in pulmonary arterial hypertension: a Delphi process and narrative review. Expert review of respiratory medicine. 2021;15(11):1493-503. doi:10.1080/17476348.2021.1931129.

Hill NS, Cawley MJ, Heggen-Peay CL. New Therapeutic Paradigms and Guidelines in the Management of Pulmonary Arterial Hypertension. Journal of managed care & specialty pharmacy. 2016;22(3 Suppl A):S3-21. doi:10.18553/jmcp.2016.22.3-a.s3.

Kim JS, McSweeney J, Lee J, Ivy D. Pediatric Cardiac Intensive Care Society 2014 Consensus Statement: Pharmacotherapies in Cardiac Critical Care Pulmonary Hypertension. Pediatric critical care medicine : a journal of the Society of Critical Care Medicine and the World Federation of Pediatric Intensive and Critical Care Societies. 2016;17(3 Suppl 1):S89-100. doi:10.1097/pcc.0000000000000622.

Klinger JR, Elliott CG, Levine DJ et al. Therapy for Pulmonary Arterial Hypertension in Adults: Update of the CHEST Guideline and Expert Panel Report. Chest. 2019;155(3):565-86. doi:10.1016/j.chest.2018.11.030.

Abman SH, Hansmann G, Archer SL et al. Pediatric Pulmonary Hypertension: Guidelines From the American Heart Association and American Thoracic Society. Circulation. 2015;132(21):2037-99. doi:10.1161/cir.0000000000000329.

Wilson M, Anguiano RH, Awdish RLA et al. An expert panel Delphi consensus statement on the use of palliative care in the management of patients with pulmonary arterial hypertension. Pulmonary circulation. 2022;12(1):e12003. doi:10.1002/pul2.12003.

McLaughlin VV, Channick R, De Marco T et al. Results of an Expert Consensus Survey on the Treatment of Pulmonary Arterial Hypertension With Oral Prostacyclin Pathway Agents. Chest. 2020;157(4):955-65. doi:10.1016/j.chest.2019.10.043.

Fukuda K, Date H, Doi S et al. Guidelines for the Treatment of Pulmonary Hypertension (JCS 2017/JPCPHS 2017). Circulation journal : official journal of the Japanese Circulation Society. 2019;83(4):842-945. doi:10.1253/circj.CJ-66-0158.

Durongpisitkul K, Sompradeekul S, Nanagara R et al. Executive summary thai pulmonary hypertension guidelines 2020. J Med Assoc Thailand. 2021;104(4):679-94. doi:10.35755/jmedassocthai.2021.04.11939.

Idrees MM, Saleemi S, Azem MA et al. Saudi guidelines on the diagnosis and treatment of pulmonary hypertension: 2014 updates. Annals of thoracic medicine. 2014;9(Suppl 1):S1-s15. doi:10.4103/1817-1737.134006.

Hsu CH, Ho WJ, Huang WC et al. 2014 Guidelines of Taiwan Society of Cardiology (TSOC) for the Management of Pulmonary Arterial Hypertension. Acta Cardiologica Sinica. 2014;30(5):401-44.

Hung CC, Cheng CC, Huang WC. 2018 TSOC guideline focused updated on diagnosis and treatment of pulmonary arterial hypertension. Journal of the Formosan Medical Association = Taiwan yi zhi. 2021;120(7):1541-2. doi:10.1016/j.jfma.2021.01.017.

Hung CC, Cheng CC, Huang WC. 2018 TSOC guideline focused updated on diagnosis and treatment of pulmonary arterial hypertension. Journal of the Formosan Medical Association = Taiwan yi zhi. 2021;120(7):1541-2. doi:10.1016/j.jfma.2021.01.017.

Park JH, Na JO, Lee JS, Kim YH, Chang HJ. 2020 KSC/KATRD Guideline for the Diagnosis and Treatment of Pulmonary Hypertension: Executive Summary. Tuberculosis and respiratory diseases. 2022;85(1):1-10. doi:10.4046/trd.2021.0022.

Zhao J, Wang Q, Wang Q et al. 2020 Chinese Expert-based Consensus on the Diagnosis and Treatment of Connective Tissue Disease Associated Pulmonary Arterial Hypertension. Rheumatology and immunology research. 2021;2(2):63-78. doi:10.2478/rir-2021-0010.

Olschewski H, Behr J, Bremer H et al. Pulmonary hypertension due to lung diseases: Updated recommendations from the Cologne Consensus Conference 2018. International journal of cardiology. 2018;272s:63-8. doi:10.1016/j.ijcard.2018.08.043.

Galiè N, Humbert M, Vachiery JL et al. 2015 ESC/ERS Guidelines for the diagnosis and treatment of pulmonary hypertension: The Joint Task Force for the Diagnosis and Treatment of Pulmonary Hypertension of the European Society of Cardiology (ESC) and the European Respiratory Society (ERS): Endorsed by: Association for European Paediatric and Congenital Cardiology (AEPC), International Society for Heart and Lung Transplantation (ISHLT). European heart journal. 2016;37(1):67-119. doi:10.1093/eurheartj/ehv317.

Humbert M, Kovacs G, Hoeper MM et al. 2022 ESC/ERS Guidelines for the diagnosis and treatment of pulmonary hypertension. The European respiratory journal. 2023;61(1). doi:10.1183/13993003.00879-2022.

Kozlik-Feldmann R, Hansmann G, Bonnet D et al. Pulmonary hypertension in children with congenital heart disease (PAH-CHD, PPHVD-CHD). Expert consensus statement on the diagnosis and treatment of paediatric pulmonary hypertension. The European Paediatric Pulmonary Vascular Disease Network, endorsed by ISHLT and DGPK. Heart (British Cardiac Society). 2016;102 Suppl 2:ii42-8. doi:10.1136/heartjnl-2015-308378.

Hansmann G, Koestenberger M, Alastalo TP et al. 2019 updated consensus statement on the diagnosis and treatment of pediatric pulmonary hypertension: The European Pediatric Pulmonary Vascular Disease Network (EPPVDN), endorsed by AEPC, ESPR and ISHLT. The Journal of heart and lung transplantation : the official publication of the International Society for Heart Transplantation. 2019;38(9):879-901. doi:10.1016/j.healun.2019.06.022.

Barberà JA, Román A, Gómez-Sánchez M et al. Guidelines on the Diagnosis and Treatment of Pulmonary Hypertension: Summary of Recommendations. Archivos de bronconeumologia. 2018;54(4):205-15. doi:10.1016/j.arbres.2017.11.014.

Fernandes C, Ota-Arakaki JS, Campos F et al. Brazilian Thoracic Society recommendations for the diagnosis and treatment of chronic thromboembolic pulmonary hypertension. Jornal brasileiro de pneumologia : publicacao oficial da Sociedade Brasileira de Pneumologia e Tisilogia. 2022;46(4):e20200204. doi:10.36416/1806-3756/e20200204.

BRAZIL. Clinical Protocol and Therapeutic Guidelines for Pulmonary Arterial Hypertension. SAS/MS Ordinance No. 35, of January 16, 2014. Available at: [https://bvsms.saude.gov.br/bvs/publicacoes/protocolo_clinico_diretrizes_terapeuticas_hipertensao_arterial_pulomonar.pdf](https://bvsms.saude.gov.br/bvs/publicacoes/protocolo_clinico_diretrizes_terapeuticas_hipertensao_arterial_pulomonar.pdf%20) . Accessed on: September 26, 2023.

Rivera-Toquica A, Saldarriaga-Giraldo CI, Echeverría LE et al. 2022 Update of the Colombian Consensus on Heart Failure with Reduced Ejection Fraction: Chapter on Heart Failure, Heart Transplantation and Pulmonary Hypertension of the Colombian Society of Cardiology and Cardiovascular Surgery. Actualización 2022 del Consenso Colombiano de Insuficiencia Cardíaca con Fracción de Eyección Reducida: Capítulo de Falla Cardíaca, Trasplante Cardíaco e Hipertensión Pulmonar de la Asociación Sociedad Colombiana de Cardiología y Cirugía Cardiovascular. 2022;29:2-. doi: <https://doi.org/10.24875/RCCAR.M22000149>.

Essop MR, Galie N, Badesch DB et al. Management of pulmonary hypertension. S Afr Med J. 2015;105(6):437-9. doi:10.7196/samj.9307.
